# In Situ Construction of n‐Type SnO_x_ Interlayer by Converting Surface Sn^4+^ for Efficient Tin Perovskite Solar Cells

**DOI:** 10.1002/advs.77051

**Published:** 2026-08-06

**Authors:** Feng Yang, Yang Yang, Kun Wang, Jingyuan Tian, Peimeng Wang, Ziyong Kang, Yang Guo, Wei Tian, Hongqiang Wang, Yu Tong

**Affiliations:** ^1^ State Key Laboratory of Solidification Processing Center for Nano Energy Materials School of Materials Science and Engineering Northwestern Polytechnical University and Shaanxi Joint Laboratory of Graphene (NPU) Xi'an People's Republic of China; ^2^ Department of Physics, Chemistry and Biology (IFM) Linköping University Linköping Sweden; ^3^ School of Integrated Circuit (School of Microelectronics) Northwestern Polytechnical University Xi'an People's Republic of China; ^4^ School of Physical Science and Technology Jiangsu Key Laboratory of Frontier Material Physics and Devices Suzhou Key Laboratory of Intelligent Photoelectric Perception Jiangsu Key Laboratory of Advanced Negative Carbon Technologies Soochow University Suzhou People's Republic of China; ^5^ Hefei National Research Center for Physical Science at the Microscale Department of Physics CAS Key Laboratory of Strongly‐coupled Quantum Matter Physics University of Science and Technology of China Hefei Anhui People's Republic of China

**Keywords:** electron extraction, enhanced stability, n‐type SnO_x_ formation, Sn^4+^ conversion, tin perovskite solar cells

## Abstract

Tin perovskites are promising lead‐free candidates for perovskite solar cells (PSCs), yet their practical application is hindered by the facile oxidation of Sn^2+^ to Sn^4+^, which induces p‐type self‐doping, defect accumulation, and progressive surface‐to‐bulk degradation. Here, we propose a novel surface treatment strategy to convert detrimental surface Sn^4+^ into a functional inorganic semiconducting interlayer, thereby significantly enhancing the efficiency and stability of tin PSCs. Tin(II) chloride dihydrate (SnCl_2_·2H_2_O) is introduced onto the as‐deposited perovskite surface, where it selectively complexes surface‐enriched Sn^4+^ via Cl^−^ coordination, enabling effective dedoping, while the supplied Sn^2+^ simultaneously fills tin vacancies. During subsequent thermal annealing, the extracted Sn^4+^ species undergo hydrolysis triggered by the released water from SnCl_2_·2H_2_O, leading to the in situ formation of an n‐type SnO_x_ layer. This process effectively suppresses non‐radiative recombination, mitigates film degradation, and promotes charge extraction. Consequently, the power conversion efficiency of tin PSCs increases from 13.21% to 16.02%, while the device retains 95% of its maximum efficiency after 75 days of storage and exhibits negligible degradation under continuous one‐sun illumination for 307 h. This work provides a new surface engineering paradigm to realize efficient and stable tin PSCs.

## Graphical abstract

An n‐type SnO_x_ layer is constructed in situ on tin perovskite surfaces by converting surface‐enriched Sn^4+^ species through a one‐step SnCl_2_·2H_2_O treatment. This strategy enhances interfacial charge extraction and suppresses defect‐induced degradation, boosting the power conversion efficiency from 13.21% to 16.02% while maintaining negligible efficiency loss over 307 h of continuous one‐sun illumination.

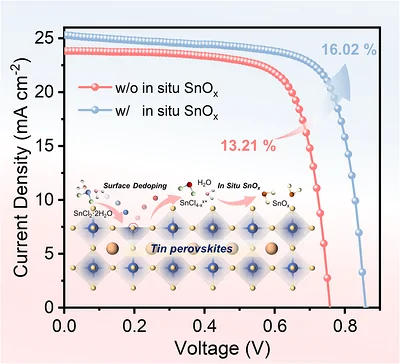

## 1 Introduction

Tin halide perovskites are among the most promising lead‐free photovoltaic materials owing to their low toxicity and near‐ideal narrow bandgap [1, 2]. However, their practical application is severely hindered by the facile oxidation of Sn^2+^, which induces p‐type self‐doping, defect accumulation, and progressive degradation of the entire film. It has been reported that such Sn^2+^ oxidation can occur even in gloveboxes with trace amounts of oxygen, and the surface of the tin perovskite film is regarded as the primarily susceptible area to oxidation due to its exposure to the environmental atmosphere. Particularly, tin perovskite solar cells (PSCs) generally adopt a p‐i‐n architecture with the surface of tin perovskite film contacting the electron transport layer (ETL), such surface Sn^2+^ oxidation induced p‐doped and defect‐rich surface gives rise to unfavorable band bending and nonradiative recombination at the perovskite/ETL interface, thereby posing huge challenges to the device efficiency and stability [3, 4, 5].

Over recent years, various strategies have been developed to suppress surface oxidation in tin perovskites, among which surface polishing or reconstruction has emerged as an effective approach for reducing surface Sn^4+^‐related defects [6, 7, 8]. For example, Wang et al. employed formamidinium chloride (FACl), which selectively coordinates with surface Sn^4+^ species to form volatile SnI_4_·xFACl complexes that can be removed during subsequent annealing, thereby achieving surface Sn^4+^ dedoping [9]. Likewise, Lu et al. introduced iso‐butylammonium iodide (iso‐BAI) together with 2‐methyl‐2‐butanol to induce a surface dissolution‐recrystallization process, reducing the surface Sn^4+^ content from 27% to 7% [10]. Although these strategies effectively reduce Sn^4+^ content and improve initial efficiency during fabrication, continuous oxygen/moisture ingress during operation can still trigger Sn^2+^ oxidation and defect formation.

To mitigate such surface‐initiated degradation, constructing protective layers on the tin perovskite surface has been explored. For example, bulky organic ligands, such as phenylethylammonium (PEA^+^), triethylammonium (TEA^+^), 4‐(aminomethyl)piperidinium (4AMP^2+^), and 1,4‐butanediammonium (BEA^2+^), can form low‐dimensional surface phases, while polymers or bulky aromatic compounds can build physical barrier layers that impede moisture and oxygen ingress [11, 12, 13]. However, these approaches tend to adversely affect electron transport between the perovskite and ETL layers due to the quantum confinement of low‐dimensional structures and the intrinsic insulating nature of long‐chain organic spacers [14, 15].

To address the interfacial charge transport issue, semiconducting interlayers have been considered to regulate the tin perovskite/ETL interface [16, 17, 18]. For instance, Li et al. introduced an n‐type organic molecule (IO‐4Cl) as an interfacial layer on tin perovskite surfaces, where the electron‐rich indacenodithieno‐[3,2‐b]thiophene backbone facilitates electron transport, thereby improving the power conversion efficiency (PCE) from 10.1% to 11.3% [19]. Similarly, Chen and co‐workers introduced the n‐type organic semiconductor NDI2HD‐Br_2_ onto the surface of tin perovskites. This interlayer modulates the surface electronic structure and promotes interfacial charge extraction, leading to a remarkable PCE increase from 10.67% to 15.01% [20]. However, organic semiconductors usually suffer from intrinsically undesirable stability, which brings potential risks for the performance of tin PSCs [21]. Even though the introduction of functional interlayers may contribute to alleviating further oxidation of Sn^2+^ from the surface, it can hardly reduce the detrimental effect of surface‐concentrated Sn^4+^, which has already been formed during film preparation. Therefore, more effective surface engineering strategies are still urgently demanded for developing efficient and stable tin PSCs.

In this work, we propose a novel and simple surface treatment strategy to make use of the detrimental Sn^4+^ at the surface of tin perovskite film and convert that to an n‐type inorganic semiconductor interlayer, thus obtaining efficient and stable tin PSCs. Specifically, surface‐enriched Sn^4+^ species are selectively complexed and displaced by Cl^−^, and the tin vacancies are favorably filled by the supplementary Sn^2+^ from Tin(II) chloride dihydrate (TCD). Furthermore, during the annealing of the tin perovskite film, water released from TCD triggers a mild hydrolysis of the extracted Sn^4+^ species, leading to the in situ formation of an n‐type SnO_x_ interlayer. These effects synergistically suppress non‐radiative recombination, facilitate interfacial charge extraction, and retard interfacial degradation. As a result, the PCE of tin PSCs increases significantly from 13.21% to 16.02%, along with markedly improved device stability. The modified device retains 95% of its peak efficiency after 75 days of storage in nitrogen, compared with 82% for the untreated device. Under continuous one‐sun illumination, it shows negligible decay after 307 h, whereas the untreated device loses over 50% of its efficiency within 70 h.

## 2 Results and Discussion

### 2.1 Conversion of Surface Sn^4+^ to an n‐Type Interlayer via TCD Treatment

It is known that the oxidation primarily occurs at the surface and gradually extends into the bulk tin perovskite films, thereby leading to the accumulation of Sn^4+^ ions and a high density of nonradiative recombination centers at the perovskite surface. These interfacial defects severely hinder efficient electron transport across the perovskite/ETL interface and deteriorate device performance [8]. TCD surface treatment addresses this issue via the following mechanisms. As shown in Figure 1a, first, due to the strong coordination affinity between Sn^4+^ and Cl^−^, the introduction of TCD onto the perovskite surface induces the preferential coordination of Cl^−^ with surface‐enriched Sn^4+^ species to form SnCl_x_ complexes, thereby facilitating the displacement of surface Sn^4+^ from the perovskite lattice [22]. Second, TCD can favorably provide supplementary Sn^2+^ to fill tin vacancies caused by Sn^2+^ oxidation. Finally, the crystalline water associated with TCD provides a localized hydrolysis environment, enabling the displaced interfacial Sn^4+^ species to gradually transform into Sn(OH)_x_ intermediates. Upon subsequent annealing, these hydroxylated species undergo thermal dehydration, leading to the in situ formation of an n‐type SnO_x_ interlayer (Note S1), which facilitates electron transport across the perovskite/ETL interface [17].

**FIGURE 1:**
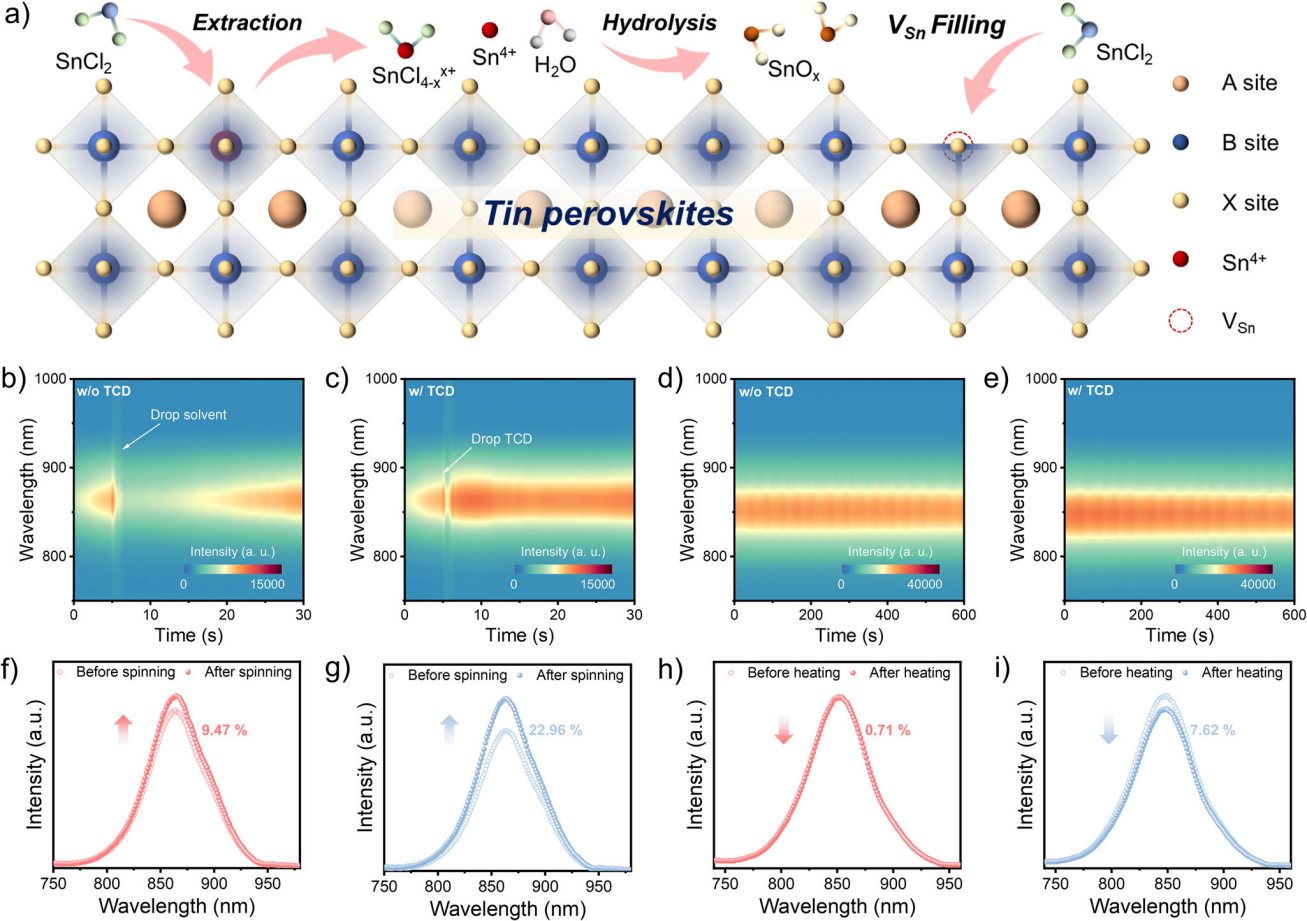
(a) Schematic illustration of TCD surface treatment on tin perovskite film. In situ PL spectra of the tin perovskites during spin‐coating (b) with pure solvent treatment, (c) with TCD treatment. In situ PL spectra of the tin perovskites during annealing (d) with pure solvent treatment, (e) with TCD treatment. Extracted PL profiles of the tin perovskites (f) with pure‐solvent and (g) TCD treatments before and after spin‐coating. Extracted PL profiles of the tin perovskites (h) with pure‐solvent and (i) TCD treatments before and after annealing.

To elucidate the interaction mechanism between TCD and tin perovskite surfaces, in situ photoluminescence (PL) spectroscopy was employed to monitor the evolution of optical properties during spin‐coating and annealing. Figure 1b,c presents the in situ PL spectra collected during spin‐coating with pure solvent (IPA/CB) and TCD‐containing solution, respectively. When the IPA/CB mixed solvent is introduced onto the tin perovskite surface approximately 5 s after the onset of spin‐coating, an immediate PL quenching is observed (5–15 s). Such quenching is generally associated with enhanced nonradiative recombination, implying the generation of charge‐trapping defect states [14]. Subsequently, the PL intensity gradually recovers with prolonged illumination from the in situ PL excitation source, which can be attributed to CB‐mediated improvements in crystal quality and the progressive filling of defect states by photogenerated carriers (15–30 s) [7]. A comparison of the PL intensities before (4 s) and after (30 s) spin‐coating (Figure 1f) reveals a modest enhancement of 9.47% following IPA/CB treatment relative to the pristine film. In contrast, no pronounced initial PL quenching is observed during TCD treatment, indicating that the surface defects generated by IPA dissolution are rapidly passivated by TCD. Furthermore, a significant PL intensity enhancement of 22.96% is achieved after TCD treatment (Figure 1g), substantially exceeding that of the solvent‐only film. This pronounced improvement clearly demonstrates that TCD effectively passivates surface defects through chemical interactions during the spin‐coating process [14].

To further probe the interfacial evolution during annealing, PL spectra recorded before and after thermal treatment were systematically compared (Figure 1d,e). Irrespective of TCD incorporation, all films exhibit a blueshift in the emission peak with increasing temperature, which can be attributed to bandgap widening induced by lattice thermal expansion [23]. The fluorescence pseudocolor images (Figure 1d,e) show that the PL intensity of the IPA/CB‐treated films changes only marginally over the 10 min annealing period, whereas the luminescence of the TCD‐treated films weakens markedly. Figure 1h,i reveals that the PL intensity of the solvent‐only film decreases by merely 0.71% after annealing, while a pronounced intensity loss of 7.62% is observed for the TCD‐treated film. This striking contrast rules out a dominant thermal quenching effect and instead points to TCD‐specific chemical processes occurring during annealing [24]. This annealing‐induced PL quenching suggests the occurrence of TCD‐related interfacial chemical evolution. Specifically, we hypothesize that crystalline water released from TCD triggers the hydrolysis of surface Sn^4+^ species and the in situ formation of an n‐type SnO_x_ layer, which is expected to enhance electron extraction at the interface and consequently quench the PL.

To verify the interfacial chemical evolution with TCD treatment, x‐ray photoelectron spectroscopy (XPS) analysis was performed. The Sn^2+^ and Sn^4+^ binding energies and their relative contents, as derived from Sn 3d XPS analysis, are summarized in Table S1. For clarity, the untreated film is denoted as the control sample, while the TCD‐treated film is referred to as the target sample. As shown in Figure 2a, the control film exhibits a high concentration of Sn^4+^ species, with the relative Sn^4+^ content exceeding 50% in both the Sn 3d_5/2_ and Sn 3d_3/2_ spectra. In contrast, the Sn^4+^ signal intensity in the target film is markedly reduced, with its total proportion decreasing to below 30%, indicating an effective reduction of surface Sn^4+^ species induced by TCD treatment. Further analysis of the binding energy shifts shows that, after TCD treatment, the Sn^4+^ 3d_5/2_ and 3d_3/2_ binding energies shift from 486.74 and 495.18 eV to 486.90 and 495.46 eV, respectively, whereas the Sn^2+^ signals remain nearly unchanged. This selective upward shift indicates an alteration in the local chemical coordination, which can be attributed to the formation of surface SnO_x_ species [25]. This is further supported by the O 1s spectra of the TCD‐treated film (Figure 2b), which exhibit a new component at 529.77 eV. This is a characteristic feature of lattice oxygen in metal oxides and is absent in pristine TCD (Figure S1), suggesting the transformation of TCD into a surface SnO_x_ layer [22, 26].

**FIGURE 2:**
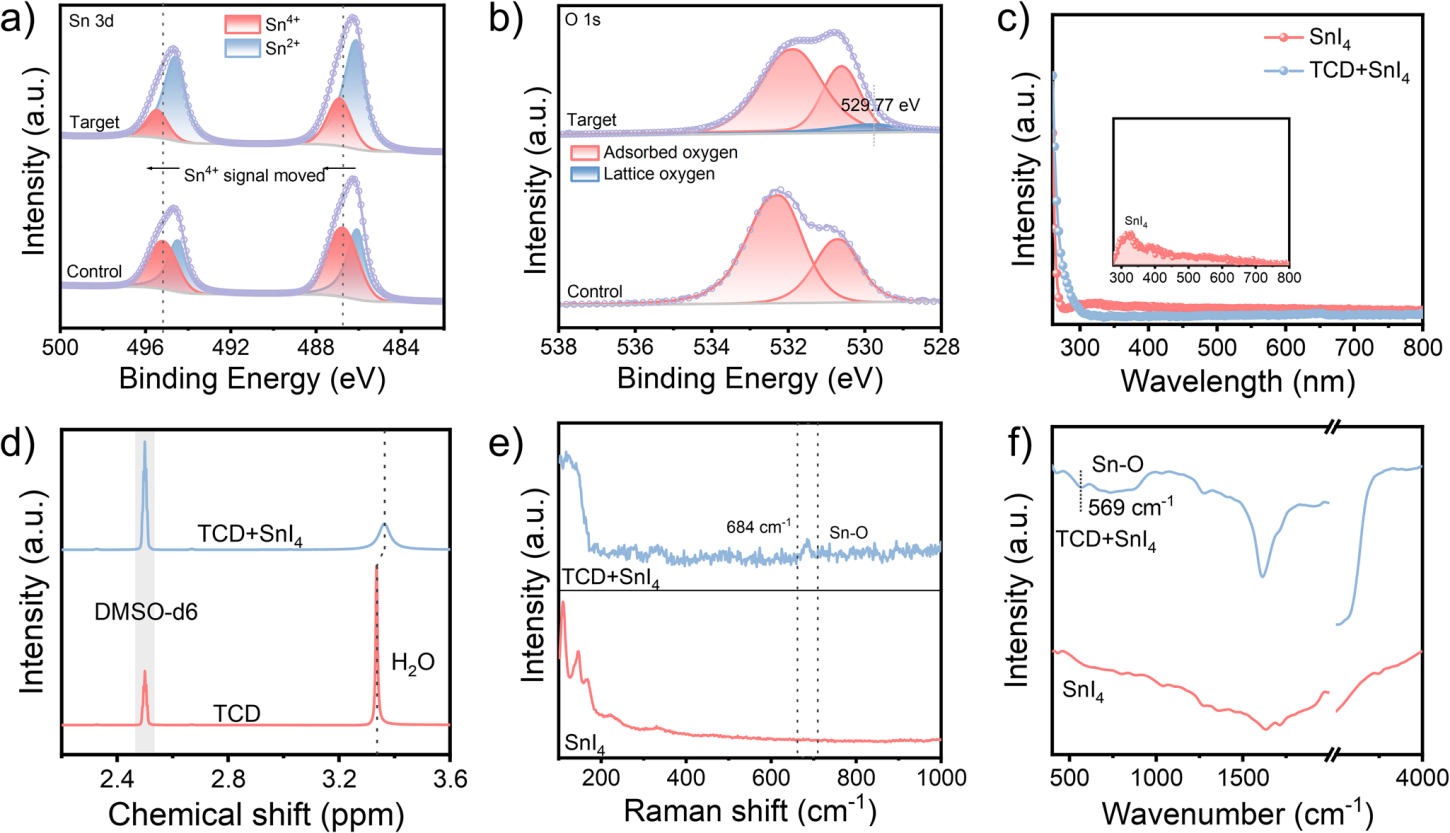
XPS spectra of (a) Sn 3d and (b) O1s for the control and target films. (c) UV–vis absorption spectra of SnI_4_, and its mixture with TCD (dissolved in DMSO; the inset shows a locally enlarged image of the SnI_4_ absorption spectrum). (d) ^1^H‐NMR spectra of TCD and its mixture with SnI_4_ (recorded in DMSO‐d6). (e) Raman spectra of SnI_4_ and its mixture with TCD. (f) FTIR spectra of SnI_4_ and its mixture with TCD.

To further clarify the spatial distribution of the TCD‐induced interfacial layer, depth‐profile XPS measurements were performed on the target films. By tracking the evolution of the O 1s signal, we found that the characteristic oxygen signal remains detectable after ∼ 1 nm of etching but disappears after ∼ 3 nm, indicating that oxygen‐containing species are confined within an ultrathin surface film with a thickness below 3 nm (Figure S2) [13]. In addition, Cl 2p depth‐profile XPS was performed to investigate the chemical state and spatial distribution of chlorine species introduced by TCD (Figure S3). Comparison of the Cl 2p spectra between pristine TCD powder and the TCD‐treated perovskite surface reveals changes in the Cl features, indicating that the precursor‐derived Cl species undergo chemical transformation during the interfacial reaction process (Figure S3a). In the near‐surface region (< 3 nm), the Cl 2p peak exhibits a shift toward lower binding energy relative to pristine TCD, suggesting a locally electron‐enriched environment for the residual Cl species (Figure S3b), which is consistent with the partial release of Cl^−^ during the hydrolysis‐driven formation of an ultrathin SnO_x_ layer (< 3 nm). At depths ≥ 3 nm, the Cl 2p peak exhibits a shift toward higher binding energy, implying the passivation of electron‐deficient defects near the surface [27]. With further increasing depth, the Cl 2p signal gradually weakens and becomes nearly undetectable at ∼ 15 nm, indicating that Cl species are primarily confined to the shallow interfacial region.

To gain insight into the chemical reactivity between TCD and Sn^4+^ species, particularly with respect to SnO_x_ formation, TCD was introduced into a SnI_4_ solution as a simplified model system. Optical images of equimolar TCD and SnI_4_ solutions show a pronounced color change from dark red to light yellow, indicating a substantial modification of the Sn^4+^ coordination environment (Figure S4). As shown in Figure 2c, the ultraviolet‐visible (UV–vis) absorption spectra further support this coordination change: the characteristic absorption peaks of SnI_4_ disappear after mixing with TCD [28, 29]. Subsequently, ^1^H NMR measurements were performed on pristine TCD and its mixture with SnI_4_ (Figure 2d). Compared with pure TCD, the characteristic resonance of water in the mixed system exhibits a pronounced downfield shift, suggesting that the strong Lewis acidity of Sn^4+^ effectively activates water molecules and may thereby trigger subsequent hydrolysis reactions [30]. The Raman spectrum (Figure 2e) of the TCD‐SnI_4_ mixed film reveals a peak in the 650–700 cm^−1^ region (centered at 684 cm^−1^), which can be assigned to characteristic Sn─O vibrations [31]. Consistently, Fourier‐transform infrared (FTIR) spectra (Figure 2f) show an absorption peak at ∼ 569 cm^−1^ in the TCD‐SnI_4_ mixture, attributed to the stretching vibration of the Sn─O bonds [32]. By contrast, these features are not observed in the pure TCD film (Figure S5), further supporting that TCD can chemically interact with Sn^4+^ species and promote the formation of Sn─O bonds. Combined with the XPS analysis of perovskite films, it is evident that TCD treatment not only induces effective surface Sn^4+^ dedoping but also promotes the in situ generation of an ultrathin SnO_x_ interfacial layer.

Building on this mechanism, the synergistic effects of surface Sn^4+^ dedoping and in situ SnO_x_ formation are expected to suppress surface degradation. To verify this, the ambient stability of the control and target films was systematically evaluated by directly exposing them to air, as shown in Figure S6. The control film exhibits pronounced color fading within ∼ 20 min of air exposure, whereas the target film exhibits discernible color changes after ∼ 90 min, demonstrating significantly enhanced ambient stability [33]. To further clarify the degradation mechanism, the in situ PL evolution of both films during air exposure was monitored (Figure S7a). For a clearer comparison, the evolution of PL intensity at a defined wavelength was extracted and normalized during the air exposure process (Figure S7b). The control film shows rapid PL quenching within the first 2 min, with its PL intensity sharply decreasing to ∼ 23% of the initial value. This behavior reflects aggravated non‐radiative recombination induced by fast Sn^2+^ oxidation and the formation of deep‐level defects. In contrast, under the same conditions, the target film retains ∼40% of its initial PL intensity, indicating improved resistance against degradation. Such enhanced stability can be ascribed to the suppression of detrimental interfacial reactions and defect formation after TCD treatment [12].

### 2.2 Regulation of Film Quality and Defect State

We evaluated the effect of TCD interface treatment on the quality of tin perovskite films. X‐ray diffraction (XRD) patterns show an enhanced (100) diffraction intensity after TCD treatment, suggesting improved crystallinity (Figure 3a and Figure S8). To probe near‐surface structural ordering, grazing‐incidence wide‐angle x‐ray scattering (GIWAXS) measurements were performed (Figure S9). The diffraction feature at q_xy_ ≈ 1 Å^−1^, assigned to the (100) plane of tin perovskite, becomes more well‐defined after TCD treatment, indicating enhanced near‐surface crystallinity [34]. Consistently, scanning electron microscopy (SEM) images (Figure 3b,c and Figure S10) show that the average grain size increases from 264 ± 71.1 nm in the control film to 311 ± 79.9 nm after TCD treatment, indicating improved crystal growth. The characteristic grain morphology of the perovskite film is well preserved after TCD treatment, consistent with the formation of an ultrathin interfacial layer. Furthermore, EDS elemental mapping (Figure S11) reveals homogeneous distributions of Cl and O throughout the target film, further supporting the uniform coverage and homogeneous interfacial modification induced by the TCD treatment.

**FIGURE 3:**
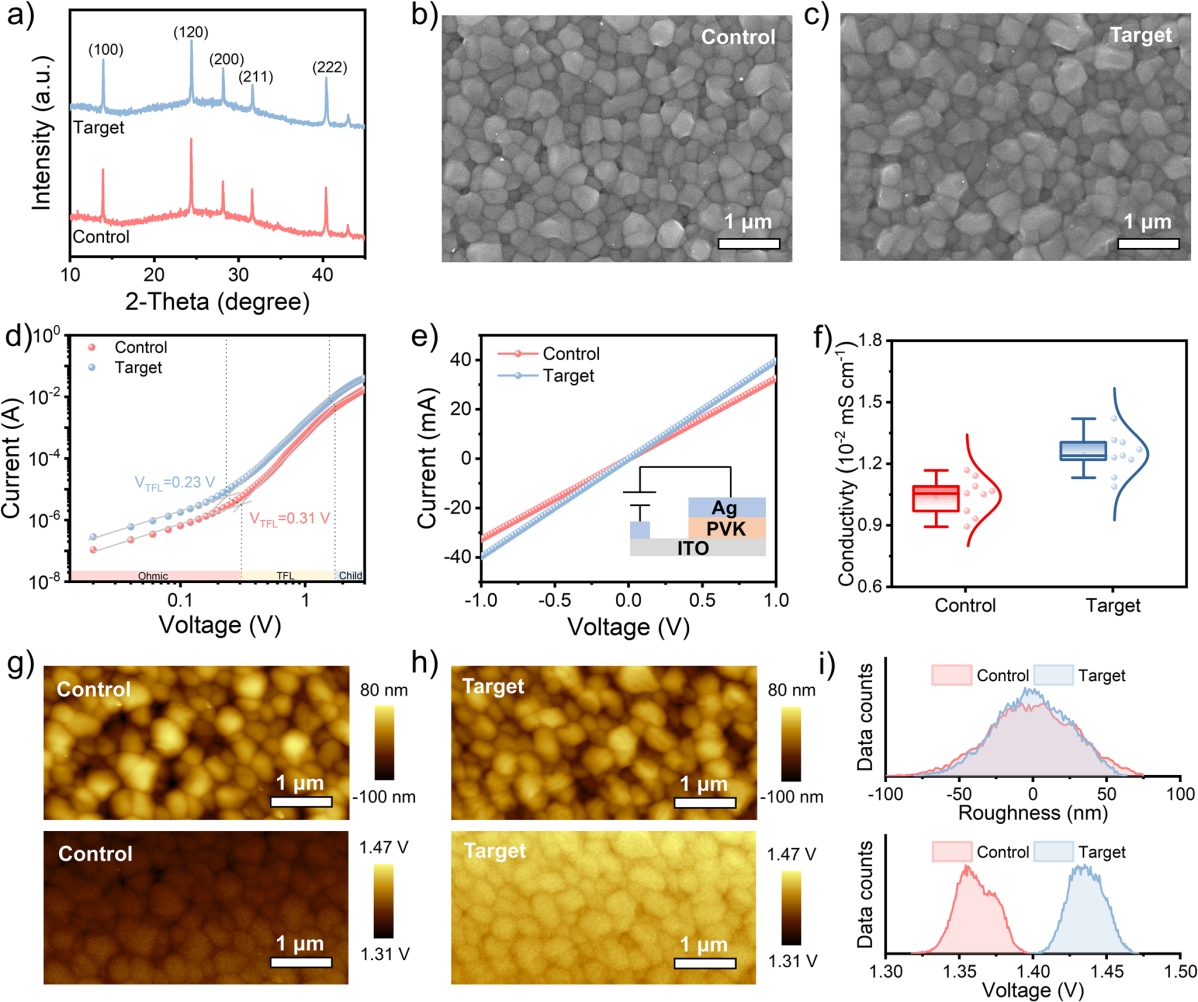
(a) XRD patterns of the control and target films. SEM images for the (b) control and (c) target films. (d) SCLC curves of electron‐only devices for the control and target devices. (e) *I‐*‐*V* curves of the control and target devices under dark conditions (inset: device structure diagram). (f) Calculated conductivity statistics of the control and target devices. AFM and KPFM images for (g) the control and (h) target films. (i) Surface uniformity and potential distribution for the control and target films.

The enhanced film quality is expected to reduce trap‐state density and improve conductivity. To quantitatively verify this effect, the defect states in the perovskite films were evaluated using electron‐only devices with a structure of ITO/SnO_2_/perovskite (with or without TCD)/C_60_/Ag configuration. Dark current‐voltage (*I–V*) measurements were performed to extract the trap‐filled limit voltage (*V_TFL_*), from which the trap‐state density (*N_t_*) was calculated according to the space‐charge‐limited current (SCLC) model, using the following equation: $N_{t}=\frac{2\varepsilon \varepsilon_{0}V_{TFL}}{qL^{2}}\;$, where ε_0_ is the vacuum permittivity, ε is the relative permittivity of the perovskite, *q* is the elementary charge, and *L* is the thickness of the perovskite layer [35]. As shown in Figure 3d, the *V_TFL_* of the electron‐only device decreases from 0.31 to 0.23 V after TCD treatment, corresponding to a reduction in electron trap density from 3.32 × 10^16^ to 2.46 × 10^16^ cm^−3^. The reduction in electron trap density suggests that TCD treatment effectively suppresses charged defect states, thereby alleviating defect‐induced carrier trapping at the perovskite interface [7]. Furthermore, we constructed ITO/perovskite (with/without TCD treatment)/Ag device structures and obtained *I–V* curves under dark conditions to evaluate the effect of TCD treatment on conductivity (Figure 3e). According to the formula: $I=\frac{\sigma A}{L}\;V$, where *A* is the effective area, the calculated conductivity of the tin perovskite film increases from 1.17 × 10^−2^ to 1.42 × 10^−2^ mS cm^−1^ after TCD treatment. This enhancement is consistently observed across nine independently fabricated subcells (Figure 3f), confirming the effectiveness of TCD in improving the electrical conductivity of tin perovskite films.

Furthermore, both the surface uniformity and electronic properties of tin perovskite films are improved after TCD interface treatment according to atomic force microscopy (AFM) and Kelvin probe force microscopy (KPFM) characterizations. As shown in Figure 3g,h, the TCD‐treated film exhibits a reduced root‐mean‐square (RMS) roughness from 29.25 to 24.97 nm, indicating a smoother overall surface, which could improve the interfacial contact between the perovskite and the ETL and promote electron extraction (Figure 3i). KPFM measurements reveal that the average contact potential difference (CPD) increases from 1.36 V for the control film to 1.44 V after TCD treatment (Figure 3i), corresponding to a reduced work function and consistent with the formation of an n‐type SnO_x_ interfacial layer. The KPFM line profiles demonstrate that the TCD‐treated film exhibits an overall increased surface potential across the entire scanned region compared with the control film, accompanied by a reduced local potential fluctuation from 35 to 31 mV (Figure S12a,b). This result indicates spatially uniform interfacial electronic modulation with improved electronic uniformity, which is beneficial for optimizing the interfacial energetic landscape, facilitating charge transport, and suppressing nonradiative recombination [36].

### 2.3 Modulation of Interfacial Carrier Dynamics

To further investigate changes in defect states and carrier transport behavior, we performed systematic optical characterizations on perovskite films before and after TCD treatment. First, PL mapping was conducted on bare perovskite films without transport layers (Figure 4a,b). Within a 70 µm × 70 µm scan area, fluorescence mapping reveals an overall suppression of the PL intensity in the target film relative to the control film. Combined with the previously demonstrated decrease in trap‐state density, this PL quenching is unlikely to arise from enhanced non‐radiative recombination. Instead, it is indicative of enhanced carrier separation and extraction at the modified surface, likely associated with the formation of a SnO_x_‐rich interfacial layer.

**FIGURE 4:**
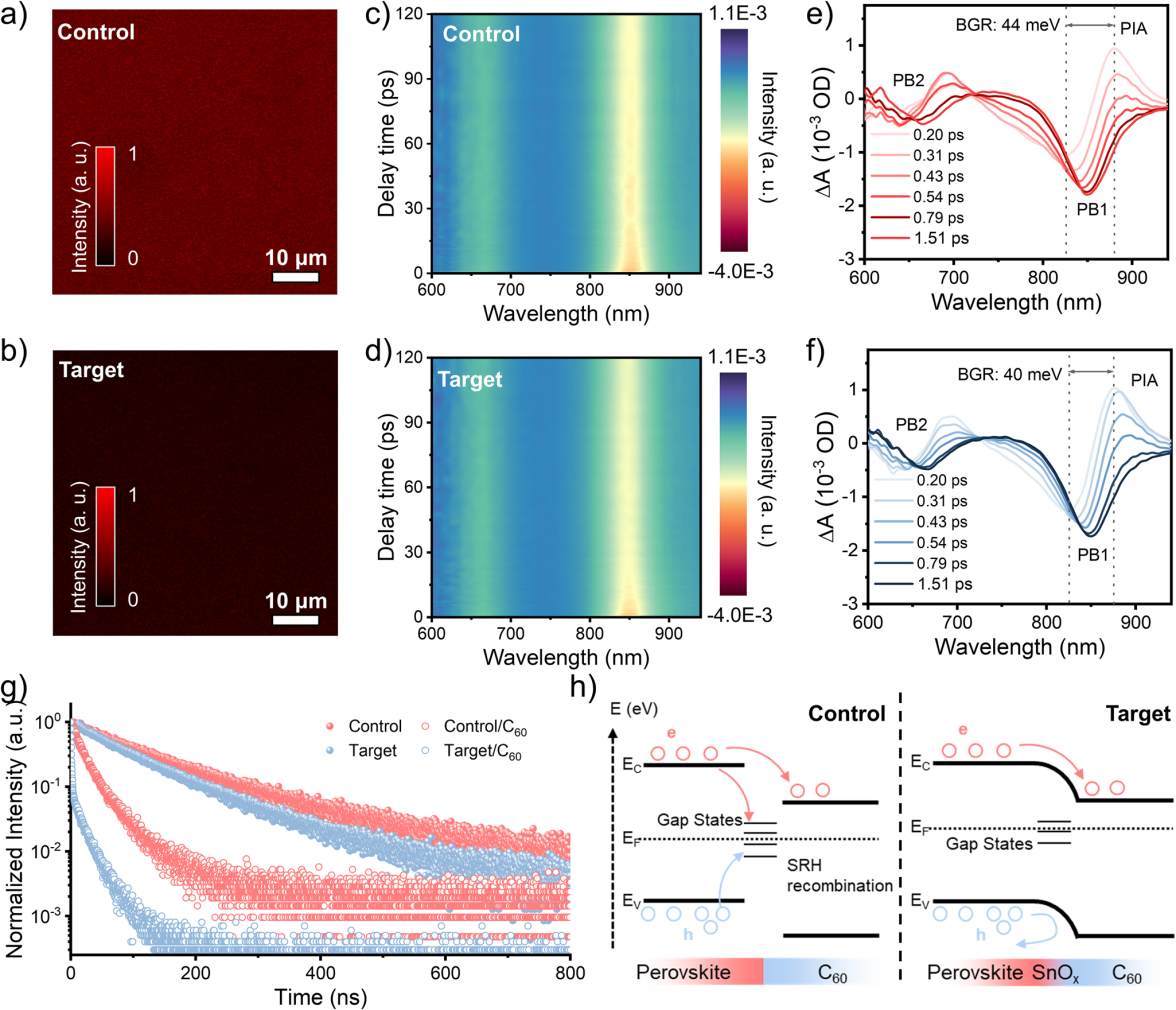
PL mapping for the (a) control and (b) target films. 2D pseudo color of TA spectroscopy for the (c) control, (d) target films. TA spectra for the (e) control and (f) target films probed at varied delays. (g) TRPL spectra of the control and target films without C_60_ and with C_60_. (h) Energy level alignment diagram for the control and target films.

Ultrafast carrier dynamics were further investigated using transient absorption (TA) spectroscopy on glass/perovskite structures with and without TCD treatment. 2D pseudocolor TA spectra are presented in Figure 4c,d, and TA spectra at selected delay times are shown in Figure 4e,f. Both samples exhibit two photobleached band (PB) signals: a dominant PB at longer wavelengths (PB1) and a weaker PB at shorter wavelengths (PB2) [37]. Additionally, photo‐induced absorption (PIA) features are also observed and are attributed to excited‐state species such as hot carriers or carriers populating the PB1 manifold [38]. Within the first picosecond after excitation, PB1 undergoes a slight redshift due to band‐filling and band‐gap renormalization effects. The band‐gap renormalization energy (BGR) for the control and target films are 40 and 44 meV, consistent with a reduced background carrier concentration resulting from TCD‐induced dedoping [39]. At longer delays (Figure S13), the target film shows a more pronounced quenching of the PB signal, reflecting accelerated electron extraction mediated by the SnO_x_ interfacial layer. Kinetic traces extracted at the PB1 maximum (Figure S14) reveal a faster carrier decay in the TCD‐treated film, providing direct evidence for enhanced interfacial charge transfer [40].

To more directly examine interfacial electron‐transport behavior, glass/perovskite (with and without TCD)/C_60_ half‐stack devices were fabricated, and steady‐state PL spectra were measured (Figure S15). Compared with the control device, the target device exhibits a pronounced PL intensity suppression, providing direct evidence for more efficient electron extraction at the perovskite/C_60_ interface after TCD treatment. In addition, a slight blueshift of the PL peak is observed in the TCD sample, which can be attributed to the suppression of localized state emission resulting from surface defect passivation. Finally, time‐resolved photoluminescence (TRPL) measurements were employed to systematically compare the carrier recombination dynamics in tin perovskite films with and without the C_60_ ETL, as shown in Figure 4g. The carrier lifetimes extracted from biexponential fitting are summarized in Tables S2 and S3. In the glass/perovskite configuration without C_60_, the average carrier lifetime decreases from 135.21 ns (control) to 103.82 ns (target), suggesting that the TCD‐induced SnO_x_ layer facilitates carrier extraction even in the absence of a dedicated ETL [41]. After depositing C_60_, both films exhibit significantly reduced lifetimes due to the efficient electron‐extracting capability of C_60_. Notably, the target film still shows a dramatically shorter lifetime (11.37 ns) than the control film (27.63 ns), indicating a synergistic effect between the TCD‐induced SnO_x_ interlayer and the C_60_ ETL in accelerating electron transfer and reducing interfacial recombination losses [42].

A full understanding of accelerated charge extraction requires correlating carrier dynamics with band structure and interfacial energy level alignment. The optical properties and band structure of the films were evaluated using UV–vis absorption spectroscopy and ultraviolet photoelectron spectroscopy (UPS). As shown in Figure S16a, the absorption is slightly enhanced after TCD treatment, which is attributed to improved film quality. Tauc plot analysis (Figure S16b,c) yields an optical bandgap of ∼ 1.40 eV for both films. UPS results and the corresponding energy‐level diagram are presented in Figures S17 and S18. It indicates that the Fermi level shifts upward from −4.52 to −4.48 eV after TCD treatment, suggesting suppressed p‐type self‐doping. This observation is consistent with the increased surface potential measured by KPFM. Meanwhile, the conduction band minimum (CBM) shifts toward better alignment with C_60_. As illustrated in Figure 4h, the reconstructed interface establishes a favorable type‐II band alignment accompanied by interfacial band bending, which facilitates electron transfer to C_60_ while suppressing hole accumulation and carrier trapping at defect sites. Collectively, these effects contribute to more efficient electron extraction and reduced interfacial recombination [43].

### 2.4 Improvement of Tin PSCs Performance

To evaluate the impact of interfacial TCD treatment on device performance, inverted tin PSCs were fabricated with the architecture ITO/PEDOT:PSS/ (Cs_0.02_(DEA_0.1_FA_0.9_)_0.98_)_0.98_EDA_0.01_SnI_3_/ ethylenediamine (EDA)/C_60_/BCP/Ag. A small amount of diethylamine hydroiodide (DEAI) was introduced as a crystallization‐regulating additive to improve film quality and reduce defect density [44]. Figure 5a displays the current density‐voltage (*J–V*) curves of the control and target devices under AM 1.5G simulated illumination. The inset of Figure 5a illustrates the device architecture, while cross‐sectional SEM images of the corresponding devices are shown in Figure S19. The control device delivers its best performance under forward scan, achieving an open‐circuit voltage (*V_OC_*) of 0.758 V, a current density (*J_SC_*) of 23.82 mA cm^−2^, a fill factor (*FF*) of 73.16%, and a *PCE* of 13.21%. In contrast, the TCD‐treated target device exhibits comprehensively improved performance: with *V_OC_* increasing to 0.859 V, *J_SC_* rising to 25.32 mA cm^−2^, FF reaching 73.65%, and the PCE markedly enhanced to 16.02%. Statistical analysis of photovoltaic parameters from 16 devices across different batches (Figure 5b and Figure S20) confirms that the TCD‐treated target devices show improved *V_OC_*, *J_SC_*, *FF*, and PCE with good reproducibility. The pronounced performance enhancement is attributed to the effective passivation of interfacial defects and the improved carrier transport efficiency enabled by TCD treatment. The reliability of the device performance was further verified through multiple approaches (Figure 5c,d). The integrated *J_SC_* values derived from the external quantum efficiency (EQE) spectra (control: 23.09 mA cm^−2^, target: 24.35 mA cm^−2^) deviate by less than 4% from the *J–V* measurements, and the stabilized power outputs (control: 13.00%, target: 15.59%) closely match the measured PCE values. To further elucidate recombination mechanisms within the devices, the dependence of *V_OC_* on light intensity was measured (Figure S21). After TCD treatment, the ideality factor decreases from 1.48 to 1.43, indicating suppressed trap‐assisted recombination [45]. Dark *J–V* measurements show that TCD treatment significantly reduces the dark current, consistent with the observed reduction in defect density (Figure S22). This conclusion is further supported by electrochemical impedance spectroscopy (EIS) measurements performed under dark conditions (Figure S23), where the target device exhibits a substantially higher recombination resistance, confirming effective mitigation of non‐radiative recombination pathways [18]. The charge transfer and carrier recombination dynamics of tin PSCs were investigated using transient photocurrent (TPC) and transient photovoltage (TPV) measurements. As shown in the TPC spectrum (Figure 5e), the target device exhibits a faster decay time (0.27 µs) compared to the control device (0.80 µs), confirming enhanced charge extraction. Figure 5f shows that the TPV decay lifetime of the target device (90.01 µs) is significantly longer than that of the control device (51.46 µs), indicating a reduction in carrier recombination, which supports the observed decrease in defect density due to the treatment. These results indicate that the treatment accelerates charge extraction while suppressing carrier recombination, thereby leading to improved photovoltaic performance. A comparison with reported C_60_‐based tin PSCs is presented in Figure 5g (Table S4), where the achieved *V*_OC_ and PCE are among the highest reported, suggesting that defect suppression and interfacial regulation help mitigate the intrinsic limitations of this widely used ETL platform.

**FIGURE 5:**
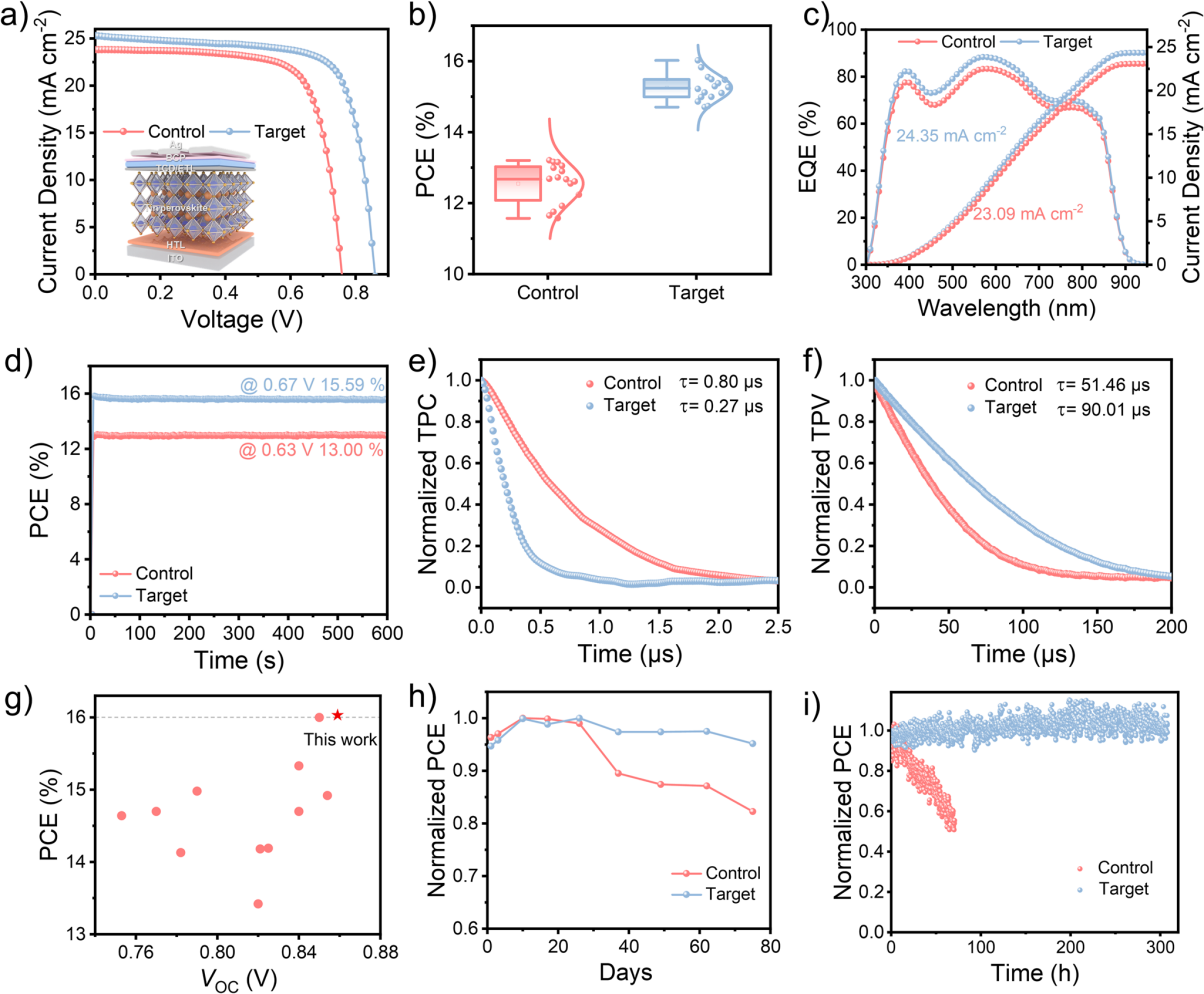
(a) *J–V* curves (inset is a schematic diagram of the tin PSCs architecture), (b) Statistical results of PCE, (c) EQE spectra, and (d) steady power outputs of the control and target devices. (e) TPC delay curves, (f) TPV delay curves of the control and target devices. (g) Statistical plot of *V*_OC_ and PCE for C_60_‐based tin PSCs. (h) Storage stability and (i) operational stability of the control and target devices.

Finally, the impact of TCD treatment on the device stability was investigated. The long‐term shelf stability of unencapsulated devices was evaluated by storing them in a nitrogen‐filled glovebox at room temperature (Figure 5h). Both device types show increased efficiency within the first 10 days of storage, likely associated with intrinsic perovskite evolution and progressive interfacial healing with EDA during early‐stage storage [46, 47]. After 75 days, the control device retains only 82% of its peak performance, whereas the target device preserves ∼ 95% under identical conditions. Furthermore, the operational stability of unencapsulated devices was evaluated under continuous one‐sun illumination (AM 1.5G, 100 mW cm^−2^) at room temperature with continuous nitrogen purging throughout the test (Figure 5i). The control device retained only ∼ 50% of its initial PCE after 70 h of operation, whereas the TCD‐treated device exhibits no noticeable performance degradation even after 307 h of continuous illumination, highlighting the pronounced enhancement in operational stability afforded by the TCD‐induced interfacial modification. This enhanced stability is consistent with the previously observed suppression of film degradation in air. The underlying mechanism is attributed to the TCD‐induced formation of an SnO_x_ layer, which effectively inhibits undesirable interfacial reactions triggered by Sn^4+^, thereby mitigating the chain reactions that lead to progressive film decomposition.

## 3 Conclusions

In this work, we address the intrinsic surface instability of tin perovskites by converting detrimental surface Sn^4+^ defects into a functional SnO_x_ interlayer via a TCD interfacial treatment. This treatment effectively reduces the surface defect density of tin perovskites, alleviates surface p‐doping, thereby suppressing non‐radiative carrier recombination and enhancing electron extraction/transport at the perovskite/ETL interface. Consequently, the PCE of tin PSCs increased from 13.21% to 16.02% after TCD treatment, accompanied by improved device storage and operational stability. After 75 days of storage in a nitrogen glove box, the PCE of the target devices remains at 95% of the highest value. Under simulated one‐sun illumination for 307 h, the device PCE shows negligible degradation. This work presents a straightforward yet adaptable approach for regulating surface doping in tin perovskites, offering valuable insights for the development of efficient and stable lead‐free perovskite photovoltaics.

## Author Contributions

**Feng Yang**: conceptualization, investigation, methodology, validation, writing original draft, formal analysis, data curation. Yang Yang: methodology, data curation, resources, validation. **Kun Wang**: conceptualization, writing – review and editing, project administration, supervision, funding acquisition. **Jingyuan Tian**: methodology, software, formal analysis, investigation. **Peimeng Wang**: methodology, software, investigation. **Ziyong Kang**: investigation, methodology, formal analysis. **Yang Guo**: methodology, software. **Wei Tian**: methodology, funding acquisition, Writing – review and editing. **Hongqiang Wang**: writing – review and editing, supervision, resources, project administration. **Yu Tong**: conceptualization, writing – review and editing, funding acquisition, project administration, supervision, resources.

## Conflicts of Interest

The authors declare no conflicts of interest.

## Supporting information




**Supporting File**: advs77051‐sup‐0001‐SuppMat.docx.

## Data Availability

The data that support the findings of this study are available from the corresponding author upon reasonable request.
