# Supplementary material for: In Situ Construction of n‐Type SnOx Interlayer by Converting Surface Sn4+ for Efficient Tin Perovskite Solar Cells
Source: Adv Sci (Weinh). 2026 Aug 6;13(60):e77051. doi: 10.1002/advs.77051 (PMC13445317; doi:10.1002/advs.77051)
Supplement: Supplementary file 1 [file ADVS-13-e77051-s001.docx]

**Supporting Information**

**In Situ Construction of n-Type SnO_x_ Interlayer by Converting Surface Sn^4+^ for Efficient Tin Perovskite Solar Cells**

*Feng Yang^1^, Yang Yang^1^, Kun Wang*^2^, Jingyuan Tian^3^, Peimeng Wang^1^, Ziyong Kang^1^, Yang Guo^4^, Wei Tian*^4^, Hongqiang Wang*^1^, Yu Tong*^5^*

^1^*State Key Laboratory of Solidification Processing, Center for Nano Energy Materials, School of Materials Science and Engineering, Northwestern Polytechnical University and Shaanxi Joint Laboratory of Graphene (NPU), Xi’an 710072, P. R. China*

^2^*Department of Physics, Chemistry and Biology (IFM), Linköping University, Linköping 58183, Sweden*

*^3^School of Integrated Circuit (School of Microelectronics), Northwestern Polytechnical University, Xi’an 710072, P. R. China*

^4^*School of Physical Science and Technology, Jiangsu Key Laboratory of Frontier Material Physics and Devices, Suzhou Key Laboratory of Intelligent Photoelectric Perception, Jiangsu Key Laboratory of Advanced Negative Carbon Technologies, Soochow University, Suzhou 215006, P. R. China*

^5^*Hefei National Research Center for Physical Science at the Microscale, Department of Physics, CAS Key Laboratory of Strongly-coupled Quantum Matter Physics, University of Science and Technology of China,* *Hefei, Anhui 230026, P. R. China.*

* Corresponding authors:

*E-mail address:*

kun.wang@liu.se

wtian@suda.edu.cn

hongqiang.wang@nwpu.edu.cn

yutong1988@ustc.edu.cn

**Experimental Section**

**Materials:** Patterned indium tin oxide (ITO, 7-9 Ω sq^-1^) glass was bought from Advanced Election Technology Co. Ltd., Formamidinium iodide (FAI, 99.5%), Diethylamine Hydroiodide (DEAI, ≥99.5%) were purchased from Greatcell Solar Materials Pty Ltd. Ethylenediamine dihydroiodide (EDAI_2_, 99.5%), Cesium iodide (CsI, 99.999%), and PEDOT: PSS aqueous solution (Clevios PVP Al 4083) were obtained from Xi'an Yuri Solar Co., Ltd. Tin(II) chloride dihydrate (SnCl_2_·2H_2_O, 98%), dimethyl sulfoxide (DMSO, ≥99.9%), N-dimethylformamide (DMF, 99.8%), 2-propanol (IPA, 99.5%), chlorobenzene (CB, 99.8%), were purchased form sigma Aldrich. Germanium (II) Iodide (GeI_2_, 99.99%), Glutathione (GSH, 98%) were obtaind from Admas. Tin (II) iodide (SnI_2_, 99.999%) was bought from 3A Materials. Tin (II) fluoride (SnF_2_, 99.9%) was supplied by Macklin. Ethylenediamine (EDA, ≥99.0%) was bought from Sinopharm Chemical Reagent Co., Ltd. Ammonium thiocyanate (NH_4_SCN, 99.99%) and Tin powder (99.5%, particle size≤5μm) were obtained from Aladdin. Phenyl-C61-butyric acid methyl ester (PCBM) was bought from Advanced Election Technology Co. Ltd. 1’,1’’,4’,4’’-tetrahydro-di[1,4]methanonaphthaleno[5,6]fullerene-C_60_ (ICBA) was purchased from 1 material. C_60_ (99%) and bathocuproine (BCP) were sourced from Vizuchem (Shanghai, China) Co., Ltd. All chemicals were used directly without further treatment.

**Device Fabrication:** The ITO glass substrates were ultrasonically cleaned sequentially with glass cleaning solution, acetone, isopropanol, and ethanol for 30 minutes each, followed by drying under nitrogen flow and plasma treatment for 15 min to improve surface wettability. Subsequently, a PEDOT:PSS layer was deposited onto the ITO substrates by spin coating at 500 rpm for 10 s and 5000 rpm for 30 s, and then annealed at 150 °C for 20 min. The substrates were then immediately transferred into a nitrogen-filled glovebox. Inside the glovebox, the perovskite precursor solution was prepared by dissolving CsI, DEAI, FAI, EDAI_2_, SnI_2_, SnF_2_, and GeI_2_ in a molar ratio of 0.0196:0.096:0.8644:0.01:1:0.1:0.05 in a mixed solvent of DMF:DMSO (4:1 by volume) to obtain a 1.0 M solution. Glutathione (GSH) and tin powder were introduced to modulate crystallization and prevent the oxidation of Sn^2+^. This precursor solution was stirred overnight and filtered before use. For perovskite film deposition, 30 µL of the filtered precursor solution was dispensed onto the PEDOT:PSS-coated substrate and spin-coated at 5000 rpm for 50 seconds (with an acceleration of 1000 rpm/s). At the 15th second after the start of the spin-coating process, 500 µL of CB was quickly dropped as an anti-solvent. After spin-coating, the wet film was immediately placed on a hotplate at 70°C and annealed for 20 minutes to form the perovskite film. For the fabrication of the target device, a solution of 0.5 mg SnCl_2_·2H_2_O (TCD) dissolved in 1 ml of a mixed solvent of IPA:CB (1:9 by volume) was prepared. Then, 30 µL of this TCD solution was dynamically spin-coated onto the perovskite film at 5000 rpm for 30 seconds, followed by annealing at 65 °C for 10 minutes. Subsequently, for both control and target devices, a solution of 0.15 mM EDA in CB was dynamically spin-coated onto the perovskite surface at 5000 rpm for 30 seconds. Immediately afterwards, a solution of PCBM:ICBA (4:1 by weight) in CB was spin-coated and annealed at 70°C for 10 minutes. Finally, the processed films were transferred to a thermal evaporation system. Once the vacuum reached 5×10^-4^ Pa, 25 nm of C_60_, 6 nm of BCP, and 100 nm of Ag were sequentially evaporated to complete the fabrication of the tin perovskite solar cell devices.

**Characterization:** The current density-voltage (J-V) curves were measured using a Keithley 2400 source meter over a voltage range of -0.1 to 0.9 V under simulated AM 1.5G solar illumination with an intensity of 100 mW cm^-2^. The scan rate was set to 0.1 V s^-1^ with a voltage step of 0.01 V. The light intensity was calibrated using a standard silicon reference cell (Oriel, VLSI Standards) prior to the measurements. The effective active area (0.05 cm^2^) was determined using an optical microscope (ZWSP-2KHD). Maximum power point (MPP) operational stability tests were conducted on unencapsulated perovskite solar cells under continuous one-sun equivalent illumination using a white LED array. The measurements were performed at 45 °C in a nitrogen atmosphere using a multi-channel solar cell stability test system (Wuhan 91PVKSolar Technology Co., Ltd., China). Electron-only devices for space-charge-limited current (SCLC) measurements were fabricated with the following structure: ITO/SnO_2_/perovskite (with or without TCD treatment)/C_60_/Ag. The I-V characteristics of these devices were measured in the dark over a voltage range of 0-4 V. Light-intensity-dependent open-circuit voltage (*V_OC_*) was extracted from J-V measurements under varying illumination intensities. Optical absorption spectra were recorded using a UV-vis spectrophotometer (UV-2700, Shimadzu). External quantum efficiency (EQE) spectra were measured using an Enli Tech EQE measurement system (QE-R3011) under dark conditions. Electrochemical impedance spectroscopy (EIS) measurement was performed using an electrochemical workstation (CHI660E, Chenhua). Transient photocurrent (TPC) and transient photovoltage (TPV) measurements were conducted using a YAG laser with a wavelength of 532 nm, a pulse width of 6 ns, and input impedance of 50 Ω and 1 MΩ, respectively. Ultraviolet photoelectron spectroscopy (UPS) measurements were carried out using a Thermo Fisher Scientific ESCALAB Xi system, while X-ray photoelectron spectroscopy (XPS) measurements were performed using a Thermo Scientific K-Alpha spectrometer. Cross-sectional and top-view scanning electron microscopy (SEM) images and corresponding energy-dispersive X-ray spectroscopy (EDS) elemental mapping were acquired using a field-emission scanning electron microscope (Sigma 300, Zeiss, Germany). *In situ* photoluminescence (PL) spectra were recorded using an Ocean Optics spectrometer equipped with a 365 nm UV LED excitation source. Time-resolved photoluminescence (TRPL) measurements were conducted using a DeltaFlex modular fluorescence lifetime system (Horiba Scientific). The average carrier lifetime was obtained through a biexponential fitting. PL mapping images were obtained using a laser scanning confocal microscope (Leica TCS SP8). ^1^H nuclear magnetic resonance (^1^H NMR) spectra were collected in DMSO-d_6_ using a Bruker 400 MHz NMR spectrometer (AVANCE NEO 400 MHz) equipped with a BBFO probe. X-ray diffraction (XRD) patterns of the films deposited on glass substrates were collected over a 2θ range of 5-45° using a Rigaku SmartLab SE diffractometer. Raman spectra were acquired using a Horiba Scientific Raman spectrometer. Grazing-incidence wide-angle X-ray scattering (GIWAXS) measurements were carried out using a Xeuss 2.0 SAXS system (Xenocs Ltd., France) at an X-ray energy of 8 keV, with a sample-to-detector distance of 110 mm and an incident angle of 0.2°. Contact potential difference (CPD) mapping and surface roughness measurements were performed using a Cypher atomic force microscope. Femtosecond transient absorption (TA) spectra were obtained using an ultrafast transient absorption spectrometer (Time-Tech Spectra, LLC).

**Note 1.**

The introduction of SnCl_2_·2H_2_O (TCD) onto the tin perovskite surface may involve a series of interfacial chemical transformations, including Sn^4+^ coordination, defect compensation, hydrolysis, and subsequent thermal conversion. The possible chemical reaction pathways are described below.[1-3]

$${Sn}_{surface}^{4+}+x{Cl}^{-}\to\left[ Sn{Cl}_{x} \right]^{\left( 4-x \right)-}$$

$${Sn}^{2+}+V_{Sn}\to{Sn}_{Sn}$$

$$\left[ Sn{Cl}_{x} \right]^{\left( 4-x \right)-}+H_{2}O\to{Sn(OH)}_{x}+x{Cl}^{-}$$

$${Sn(OH)}_{x}\to{SnO}_{x}+H_{2}O$$

**Fig. S1.** XPS spectra of O 1s for pristine TCD.

**Fig. S2.** Evolution of O 1s XPS spectra of TCD-treated tin perovskite films during depth-profiling etching.

**Fig. S3.** a) Comparison of the Cl 2p spectra of pristine TCD and the target film. b) Evolution of Cl 2p XPS spectra of TCD-treated tin perovskite films during depth-profiling etching.

**Fig. S4.** Optical images of SnI_4_, TCD, and their mixtures dissolved in a DMF/DMSO (4:1) solvent system.

**Fig. S5.** a) Raman spectra and b) FTIR spectra of TCD.

**Fig. S6.** Air stability of the control and target films.

**Fig. S7.** In situ PL spectra of the control and target films during air exposure: a) Time-evolution of PL spectra. b) Normalized PL intensity at ~862 nm versus time.

**Fig. S8.** Comparison of the diffraction peak intensity of (100) crystal planes between the control and target films.

**Fig. S9.** 2D GIWAXS patterns of the a) control and b) target films.

**Fig. S10.** Statistical analysis of grain size for the a) control and b) target films.

**Fig. S11.** EDS elemental mapping for the target film, a) Cl element and b) O element.

**Fig. S12.** a) KPFM images for the control and target films, and b) corresponding potential profiles.

**Fig. S13.** At longer delays: 2D pseudo color plot of TA spectroscopy for the a) control, b) target films. TA spectra of the c) control and d) target film probed at different delays.

**Fig. S14.** TA signal decay traces for the control and target films.

**Fig. S15.** PL spectra of the control and target films with a C_60_ transport layer.

**Fig. S16.** a) UV‑Vis absorption spectra for the control and target films, Tauc plots for the b) control and c) target films.

**Fig. S17.** UPS results for the control and target films.

**Fig. S18.** Band alignment between perovskite and transport layer.

**Fig. S19.** Cross-sectional SEM images for the a) control, b) target films.

**Fig. S20.** Statistical results of a) *V*_OC_, b) *J*_SC_, c) *FF* for the control and target devices.

**Fig. S21.** Light intensity dependent *V_OC_* for the control and target devices.

**Fig. S22.** Dark J-V curves for the control and target devices.

**Fig. S23.** EIS curves for the control and target devices.

**Table S1.** Analysis of Sn^2+^ and Sn^4+^ contents of tin perovskite films with and without surface treatment.

| **Sample** | **Sn 3d5/2** | | | **Sn 3d3/2** | | |
| --- | --- | --- | --- | --- | --- | --- |
|  | Sn^2+^ (eV) | Sn^4+^ (eV) | Sn^2+^/Sn^4+^ (%) | Sn^2+^ (eV) | Sn^4+^ (eV) | Sn^2+^/Sn^4+^ (%) |
| **Control** | 486.05 | 486.74 | 55.35 | 494.47 | 495.18 | 51.55 |
| **Target** | 486.09 | 486.90 | 29.38 | 494.57 | 495.46 | 21.57 |

**Table S2.** Fitting parameters of the TRPL spectra for tin perovskite films with and without surface treatment.

| **Sample** | **A_1_ (%)** | **τ_1_ (ns)** | **A_2_ (%)** | **τ_2_ (ns)** | **τ_avg_ (ns)** |
| --- | --- | --- | --- | --- | --- |
| Control | 59.15 | 77.60 | 40.85 | 172.69 | 135.21 |
| Target | 70.79 | 69.33 | 29.21 | 144.04 | 103.82 |

**Table S3.** Fitting parameters of the TRPL spectra for tin perovskite films with and without surface treatment.

| **Sample** | **A_1_ (%)** | **τ_1_ (ns)** | **A_2_ (%)** | **τ_2_ (ns)** | **τ_avg_ (ns)** |
| --- | --- | --- | --- | --- | --- |
| Control | 71.00 | 13.25 | 29.00 | 39.46 | 27.63 |
| Target | 93.67 | 0.79 | 6.33 | 18.18 | 11.37 |

**Table S4.** Summary of photovoltaic parameters for devices based on a C_60_ transport layer.

| **Configuration** | ***V*_OC_ (V)** | ***J*_SC_ (mA/cm^2^)** | | **FF (%)** | **PCE (%)** | **Ref.** |
| --- | --- | --- | --- | --- | --- | --- |
| ITO/NiO_x_/2PADBC/ perovskite /C_60_/BCP/Ag | 0.825 | | 23.23 | 74.06 | 14.19 | [4] |
| ITO/ EMIC-PEDOT:PSS / perovskite /ICBA/C_60_/BCP/Ag | 0.79 | | 23.86 | 79.45 | 14.98 | [5] |
| ITO/PEDOT:PSS/perovskite /C_60_/BCP/Ag | 0.77 | | 24.90 | 76.7 | 14.70 | [6] |
| ITO/PEDOT:PSS/perovskite/ C_60_/BCP/Ag | 0.821 | | 24.32 | 0.74 | 14.18 | [7] |
| ITO/PEDOT:PSS/perovskite/ C_60_/BCP/Ag | 0.753 | | 25,83 | 75.3 | 14.64 | [8] |
| ITO/PEDOT:PSS/perovskite/ C_60_/BCP/Cu | 0.782 | | 22.76 | 78.46 | 14.13 | [9] |
| ITO/PEDOT:PSS/perovskite/ C_60_/BCP/Ag | 0.84 | | 24.72 | 73.61 | 15.33 | [10] |
| ITO/PEDOT:PSS/2PADCB/  perovskite/ C_60_/BCP/Cu | 0.84 | | 23.5 | 74.8 | 14.7 | [11] |
| ITO/ PEDOT:PSS/ perovskite/ C_60_/BCP/ Ag | 0.82 | | 21.64 | 73.6 | 13.42 | [12] |
| ITO/PEDOT:PSS/perovskite/C_60_/  Yb(acac)_3_/Cu | 0.854 | | 23.39 | 74.69 | 14.92 | [13] |
| ITO/EMIC-PEDOT:PSS/perovskite/ICBA/ C_60_/BCP/Ag | 0.85 | | 23.89 | 78.79 | 16.00 | [14] |
| ITO/PEDOT:PSS/perovskite/ C_60_/BCP/Ag | 0.859 | | 25.31 | 73.65 | 16.03 | This work |

**Reference**

[1] Song, J.W., Shin, Y.S., Kim, M., et al. Post-Treated Polycrystalline SnO_2_ in Perovskite Solar Cells for High Efficiency and Quasi-Steady-State-IV Stability. Advanced Energy Materials 14 (2024). 2401753. https://doi.org/10.1002/aenm.202401753.

[2] Guan, X., Li, Y.Q., Meng, Y.Y., et al. Targeted elimination of tetravalent-Sn-induced defects for enhanced efficiency and stability in lead-free NIR-II perovskite LEDs. Nature Communications 15 (2024). 9913. https://doi.org/10.1038/s41467-024-54160-x.

[3] Hu, M.Y., Zhang, Y.L., Gong, J., et al. Surface Sn(IV) Hydrolysis Improves Inorganic Sn-Pb Perovskite Solar Cells. ACS Energy Letters 8 (2023). 1035-1041. https://doi.org/10.1021/acsenergylett.2c02435.

[4] Li, B., Zhang, C., Gao, D., et al. Suppressing Oxidation at Perovskite-NiOx Interface for Efficient and Stable Tin Perovskite Solar Cells. Advanced Materials 36 (2024). 2309768. https://doi.org/10.1002/adma.202309768.

[5] Zhou, X., Peng, W., Liu, Z., et al. Additive engineering with 2,8-dibromo-dibenzothiophene-S, S-dioxide enabled tin-based perovskite solar cells with 14.98% power conversion efficiency. Energy & Environmental Science 17 (2024). 2837-2844. https://doi.org/10.1039/d3ee03359g.

[6] Zhou, J.H., Hao, M.W., Zhang, Y., et al. Chemo-thermal surface dedoping for high-performance tin perovskite solar cells. Matter 5 (2022). 683-693. https://doi.org/10.1016/j.matt.2021.12.013.

[7] Zhang, Z., Liu, J., Bi, H., et al. Over 14% efficiency of highly reproducible Sn perovskite solar cell via defect passivation and morphology repairment. Chemical Engineering Journal 483 (2024). 149345. https://doi.org/10.1016/j.cej.2024.149345.

[8] Wang, L., Miao, Q.Q., Wang, D.D., et al. Ternary Passivation for Enhanced Carrier Transport and Recombination Suppression in Highly Efficient Sn-Based Perovskite Solar Cells. Small 20 (2024). 2404026. https://doi.org/10.1002/smll.202404026.

[9] Jin, J.L., Zhang, Z.H., Zou, S.L., et al. Regulating Compressive Strain Enables High-Performance Tin-Based Perovskite Solar Cells. Advanced Energy Materials 15 (2025). 2403718. https://doi.org/10.1002/aenm.202403718.

[10] Wang, L., Bi, H., Liu, J., et al. Exceeding 15% Performance with Energy Level Tuning in Tin-Based Perovskite Solar Cells. ACS Energy Letters 9 (2024). 6238-6244. https://doi.org/10.1021/acsenergylett.4c03172.

[11] Qu, J.Y., Wang, X.X., Luo, C., et al. Enhancing Charge Collection of Tin-Based Perovskite Solar Cells by Optimizing the Buried Interface with a Multifunctional Self-Assembled Monolayer. ACS Applied Materials & Interfaces 17 (2025). 19783-19794. https://doi.org/10.1021/acsami.5c01653.

[12] Jung, S., Jang, Y., Jung, H., et al. Radical scavenger-driven oxidation prevention and structural stabilization for efficient and stable tin-based perovskite solar cells. Energy & Environmental Science 18 (2025). 6076-6084. https://doi.org/10.1039/d5ee00735f.

[13] Wang, X.X., Zeng, C.W., Yang, Z.H., et al. Ytterbium Acetylacetonate as Cathode Buffer Layer for Tin-Based Perovskite Solar Cells. Advanced Functional Materials 36 (2026). e75080. https://doi.org/10.1002/adfm.75080.

[14] Zhou, X.Y., Wang, Y.W., Huang, K.X., et al. A Multifunctional Reducing Molecular Dopant for 16% Efficient Tin Perovskite Solar Cells. Rare Metals 45 (2026). e70162. https://doi.org/10.1002/rar2.70162.
